# Entropy and Mutability for the *q*-State Clock Model in Small Systems

**DOI:** 10.3390/e20120933

**Published:** 2018-12-06

**Authors:** Oscar A. Negrete, Patricio Vargas, Francisco J. Peña, Gonzalo Saravia, Eugenio E. Vogel

**Affiliations:** 1 Departamento de Física, Universidad Técnica Federico Santa María, Valparaíso 2340000, Chile; 2 Centro para el Desarrollo de la Nanociencia y la Nanotecnología, CEDENNA, Santiago 8320000, Chile; 3 Departamento de Ciencias Físicas, Universidad de La Frontera, Temuco 4811230, Chile

**Keywords:** *q*-state clock model, entropy, Berezinskii–Kosterlitz–Thouless transition

## Abstract

In this paper, we revisit the *q*-state clock model for small systems. We present results for the thermodynamics of the *q*-state clock model for values from q=2 to q=20 for small square lattices of L×L, with L ranging from L=3 to L=64 with free-boundary conditions. Energy, specific heat, entropy, and magnetization were measured. We found that the Berezinskii–Kosterlitz–Thouless (BKT)-like transition appears for q>5, regardless of lattice size, while this transition at q=5 is lost for L<10; for q≤4, the BKT transition is never present. We present the phase diagram in terms of *q* that shows the transition from the ferromagnetic (FM) to the paramagnetic (PM) phases at the critical temperature $T_{1}$ for small systems, and the transition changes such that it is from the FM to the BKT phase for larger systems, while a second phase transition between the BKT and the PM phases occurs at $T_{2}$. We also show that the magnetic phases are well characterized by the two-dimensional (2D) distribution of the magnetization values. We made use of this opportunity to carry out an information theory analysis of the time series obtained from Monte Carlo simulations. In particular, we calculated the phenomenological mutability and diversity functions. Diversity characterizes the phase transitions, but the phases are less detectable as *q* increases. Free boundary conditions were used to better mimic the reality of small systems (far from any thermodynamic limit). The role of size is discussed.

## 1. Introduction

The *q*-state clock model is the discrete version of the famous 2D XY model, which is probably the most extensively studied example showing the Berezinskii–Kosterlitz–Thouless (BKT) transition [1,2,3,4] in the presence of a frustrated quenched disordered phase [5,6,7,8]. The *q*-state clock model is often used as a reference model due to its peculiar critical behavior at the transition point and its universal features [9,10,11,12,13]. Instead of the exclusion of the explicit continuous symmetry essential for the BKT transition, it can also emerge from a system without explicit continuous symmetry [13]. The Hamiltonian of the *q*-state clock model can be written in many forms, and one of the simplest forms is the following expression, where no magnetic anisotropies or external magnetic fields are included:

(1) $$H=-\frac{J}{2}\sum_{<i,j>}\cos\left(\theta_{i}-\theta_{j}\right)\;,$$

where J>0 is the ferromagnetic coupling connecting pairs of nearest neighbors *i* and *j*; the discrete angle between the spin orientations is given by $\theta_{i,j}\left(\eta ,q\right)=2\pi \eta /q$, for η={0,1,…,q−1}. While the exact XY model is recovered only in the limit of infinite *q*, it has been found that the BKT characteristics appear in the clock models when q≥5 [14,15,16,17,18,19]. The nature of the phase transitions in the general clock model has been widely studied with different theoretical and numerical approaches. However, these studies have given mixed results for the characterization of transitions at the lower bound of *q* (for instance, see the summary of the related debates in [20]).

In the present work, we want to consider the clock model as a generalization of the Ising model by establishing the similarities and differences that arise due to the increase of the degrees of freedom due to the local states at each site. All of the characterizations are aimed at the behavior of small systems: specifically, we focus on square lattices ranging from 3 × 3 up to 64 × 64. Free boundary conditions are preferred since they better represent the importance of surface states in small systems. The clock model systems under scrutiny range from q=2 (equivalent to the usual Ising model) to q=20.

There are various scattered results for the thermodynamics and phase transition of the clock model. Therefore, we report below a consistent compendium of its thermodynamic properties, such as internal energy, *U*; specific heat, *C*; entropy *S*; and magnetization. We also report the transition temperatures $T_{1}$ for transitioning from the ferromagnetic (FM) to the *P* phase for very small *q* values, continuing to the transition from the FM to the BKT phase for larger values of *q*, as well as $T_{2}$ for the transition from the BKT phase to the disordered paramagnetic (P) phase. These series of results are elaborated into a phase diagram of $T_{C}$ vs. *q*, where $T_{C}$ is determined from the C(T) curves.

The information content of a sequence was measured by the mutability, called as such for the first time by Vogel et al. [21] in a relationship with the characterization of the 2D phase transitions in the Edwards–Anderson spin system as an alternative to the Binder cumulant analysis [22]. Later, an appropriate information recognizer was proposed (named word length zipper (wlzip)) which optimizes recognition of digital information associated with properties of the system [23]. The method was later successfully applied to the reentrant phase diagram in the case of the 3D Edwards–Anderson model [24]. Successive applications of the information content methods addressed stock markets [25], pension funds [26], blood pressure [27], seismology [28], nematic transitions [29], and wind energy production [30].

In the present paper, we follow the approach of magnetic transitions [21,23] complemented with the definitions of mutability and diversity [25], to be elaborated below. The aim is to establish an alternative method to characterizing and distinguishing the phases present in the q clock model.

In the next section, we present the clock model and the main methods used to characterize it. A presentation of the results and discussion are given in Section 3, and the last section is devoted to conclusions.

## 2. Model and Methods

### 2.1. General Definitions

Let us begin by considering the *q*-state clock model on a two-dimensional (2D) square lattice of dimensions L×L=N, where the local magnetic moment or “spin” $S_{i}$ at site *i* can point in any of the *q* directions in a given plane. $S_{i}$ is then a 2D vector, i.e., $S_{i}=\left(\cos\left(\frac{2\pi }{q}k\right),\sin\left(\frac{2\pi }{q}k\right)\right)$, where k=0,1,…,q−1, with equal probability for all *q* values. $S_{i}$ are dimensionless vectors of magnitude one.

The isotropic Hamiltonian for such a system can be written as:

(2) $$H=-\frac{J}{2}\sum_{<i,j>}^{N}S_{i}\cdot S_{j}-B\cdot \sum_{i}S_{i}\;,$$

where J>0 is the ferromagnetic exchange interaction to nearest neighbors *i* and *j*; the sum extends to all such pairs through the lattice, which is indicated by the symbol <i,j> under the summation symbol. *B* is an external field applied along one direction in the plane.

In this Hamiltonian, *J* is one unit of energy and *B* is also measured in energy units. This form is completely equivalent to the one of Equation (1), when B=0.

### 2.2. Exact Theoretical Approach for a Small System

Let us begin by considering the theoretical approach for the *q*-state clock lattice with L=3, which was introduced in the previous section. The partition function can be expressed as:

(3) $$Z\left(T,B\right)=\sum_{n=1}^{\lambda }c_{n}\;e^{-E_{n}/T}\;,$$

where the coefficients $c_{n}$ are the numbers of all of the possible spin configurations compatible with energy $E_{n}$ according to the Hamiltonian of Equation (2); in the case of a magnetic field *B*, the energies and the coefficients $c_{n}$ depend upon *B*; λ is the number of different energy levels. We express energy and temperature in the same units, so the Boltzmann constant is set to unity, $k_{B}=1$.

The coefficients $c_{n}=c\left(E_{n},q\right)$ for a small N=L×L=3×3 lattice can be straightforwardly calculated by computing the energies for all $q^{9}$ spin configurations, which are shown in Table 1 for q=4 and q=6 and in Table 2 for q=5 as examples. For all even *q* values (as in the examples in Table 1), some symmetry rules apply: energy distribution is symmetric around $E_{n}=0$, and the majority of the density of states occur for $E_{n}=0$ and $c\left(-E_{n},q\right)=c\left(E_{n},q\right)$ holds. There are $q^{9}$ states to be spread among all available energies in the energy range $E_{n}=\left[-12,12\right]$. Due to symmetry, the total number of different energies are λ=11, 23, 47, 289, and 699 for q=2, 4, 6, 8, and 10, respectively.

For odd *q* values, the main symmetry of the Hamiltonian of Equation (2) for B=0 is lost, because the spin inversion $S_{i}\to -S_{i}$ is not possible. This is simply because, for odd *q* values, if there is the symmetry $S_{i}$, then $-S_{i}$ does not exist. Therefore, the energy distribution and the corresponding degeneracy is not symmetric around $E_{n}=0$. Thus, the highest possible energy is not the negative value of the ground state energy. However, as we will see, this lowering of symmetry does not affect the thermodynamic observables.

Once the partition function is known, the thermodynamic observables can be calculated directly. Thus, for the cases of internal energy *U*, specific heat *C*, and entropy *S*, they can be obtained by employing:

(4) $$U\left(T\right)=T^{2}\frac{\partial }{\partial T}\ln Z\left(T,B\right)\;,$$

(5) $$C\left(T\right)=\frac{\partial U}{\partial T}\;,$$

(6) $$S\left(T\right)=\frac{U}{T}+\ln Z\left(T,B\right)\;.$$

The next table shows the results for q=5, where the energies $E_{n}$ and degeneracies for n=1 to n=85 are explicitly given. In this case, for a given energy $E_{n}$, their negative counterpart does not exist.

We now use numeric simulations to calculate larger sizes for the same system.

### 2.3. Numerical Simulations

In addition to the theoretical calculations, most of the work presented here deals with numerical calculations based on Monte Carlo (MC) simulations. A square lattice L×L was chosen; free boundary conditions were imposed; a site was randomly visited, and the energy cost, Δ, of rotating the corresponding spin around *q* possible states was calculated: if the energy is lowered, the change of orientation is accepted; otherwise, only when exp(−Δ/T)≤r is the spin rotation accepted, where *r* is a freshly generated random number in the range [0,1] with equal probability. This is the usual Metropolis algorithm. A Monte Carlo step (MCS) is reached after N=L×L spin-rotation attempts. One of the main goals here is to report the sensitive temperatures, $T_{1}$ and $T_{2}$, that define the transition from FM to BKT and from BKT to PM (disordered) phase for different systems.

For each lattice size and q value, a sequence of temperatures was defined in the range [0.02,3] at steps of 0.02 for each temperature. Also, 5τ MCSs were performed: the first τ MCS was used to equilibrate at a fixed temperature *T*, while the next 4τ MCS was used to measure the observables every 20 MCSs, reaching a total of $2\times 10^{5}=200,000$ measurements. Unless otherwise specified, $\tau =10^{6}$ is used in the rest of the paper; this τ value gives stable results and leads to coincidence with the analytic expressions obtained as described above. The energy U(T) was computed according to the Hamiltonian given by Equation (2). With the internal energy known as a function of *T*, Equations (4)–(6) can be used to generate the thermodynamics. In parallel, the magnetization M(T) for each temperature can also be instantaneously measured. Alternatively, we can also use direct relationships based on the thermal treatment of the variables, as presented next.

### 2.4. Thermal Averages

The lattice average of the spin configuration, equivalent to the magnetization per site *M*, is given by the following expression:

(7) $$M=\frac{1}{N}\sum_{j=1}^{N}S_{j}\;,$$

where $S_{j}$ is the value of the spin at site *j* at a given time *t*, and N=L×L is the total number of spins. In this particular case, *M* is a vector of two components, $M=(M_{x},M_{y})$. Normally, the magnitude or absolute value of this vector is calculated, i.e., $\left|M\right|=\sqrt{M_{x}^{2}+M_{y}^{2}}$. Then, the thermal average of the absolute value |M| is <|M|>, and it is given by

(8) $$<|M|>=\frac{1}{N_{c}}\sum_{i=1}^{N_{c}}\sqrt{M_{x}^{2}+M_{y}^{2}}\;,$$

where $N_{c}=2\times 10^{5}$ is the number of configurations used to perform thermal averages, as explained in the preceding section.

Energy is the main value used in the Monte Carlo method to reach thermal equilibrium. Therefore, after τ MCSs, the internal energy *U* can be obtained by averaging the $N_{c}=2$ values for $E_{k}$, where *k* runs over the accepted configurations after the Metropolis algorithm, namely:

(9) $$U=<E>=\frac{1}{N_{c}}\sum_{k=1}^{N_{c}}E_{k}\;,$$

where every spin configuration is separated from the next one by 20 MCs. The energy per site is then U/N, which is the thermal average of the lattice average of the system energy.

The specific heat is then calculated as proportional to the fluctuations of the energy as follows:

(10) $$\begin{array}{cc} C & =\frac{\langle E^{2}\rangle -{\langle E\rangle }^{2}}{T^{2}}\;\;, \end{array}$$

(11) $$\begin{array}{cc} C & =\frac{1}{T^{2}}\left[\left(\frac{1}{N_{c}}\sum_{k=1}^{N_{c}}E_{k}^{2}\right)-{\left(\frac{1}{N_{c}}\sum_{k=1}^{N_{c}}E_{k}\right)}^{2}\right]\;\;. \end{array}$$

The absolute entropy *S* can be calculated by calculating $\Delta S\left(T_{f},T_{i}\right)=S\left(T_{f}\right)-S\left(T_{i}\right)$ by numerical integration of the specific heat divided by the temperature, as follows:

(12) $$\Delta S\left(T_{f},T_{i}\right)=\int_{T_{i}}^{T_{f}}\frac{C(T)}{T}dT\;.$$

We know the entropy at zero temperature, because we know the energy degeneration at T=0. For the case B≠0, only one spin configuration has minimum energy (every spin aligned to *B*); therefore, S(0)=0. On the other hand, when the magnetic field is zero, there are *q* ferromagnetic spin configurations with equal energies. Therefore, S(0)=lnq and, hence, S(T)=lnq+ΔS(T,0).

As we have seen, the thermal average of a physical quantity is a summation over $N_{c}$ quantities (normalized to $N_{c}$), like energies, magnetic moments, etc. However, the order of the sequence of these $N_{c}$ quantities is totally irrelevant to the result of this evaluation. Next, we introduce the mutability, which is a quantity (defined in terms of information theory) that can be calculated from any temporal sequence of quantities, like energy, magnetic moment, etc. Mutability is a quantity that depends on the sequence order, and, therefore, it contains information that differs from a mere average.

### 2.5. Information Theory, Mutability, and Diversity

Word length zipper (wlzip for short) is a compressor designed to recognize meaningful information in a data chain. As its name indicates, it recognizes “words” of precise length and precise location within the data chain. Then, it compresses less than other file compressors which precisely optimize this function. In the case of wlzip, the optimization is of the recognition of patterns beginning at a precise location and for a given number of digits. In this way, physical properties and/or other inherent properties of the system can be recognized.

The recognition of information within a file can render at least two parameters: mutability and diversity. They were defined a few years ago, including a working example, given in Table 2 of Reference [23]. Here, we very briefly review these definitions, beginning with the basic rules under which wlzip operates. Let us assume our data are contained in a vector file (one register per row) named “data.txt” whose number of rows is λ(data) and whose weight in bytes is w(data). We now create a map of the previous file in the following way: we go over each row of data.txt and, whenever this value is new, we add it as a new row to a new file which will be named map.txt. So, the process begins with the first element of data.txt which is also the first element in map.txt. Then, the second element of data.txt is considered: if this element is new, we add it as a second row in map.txt; if this element is the same as the previous one, we just add a digit 1 to the right of this element in map.txt (here, 1 means one position from last time this element appeared). The process continues like that so that each time a new record is detected, a new row in map.txt is created. This register is copied in map.txt at the beginning of a new row, and the first number that is written to its right is its absolute position in the data.txt file. Each time a register is repeated, its position with respect to its last appearance in the data.txt file is written to the right of the last annotation on this row in the map file. At the end of the process, map.txt contains as many rows as there were different values present in data.txt; the more a value is repeated, the wider the corresponding row is in map.txt. In a sense, this is like a histogram organized according to the appearance order. The number of rows in map.txt is $\lambda^{*}\left(data\right)$, while the weight of this file is $w^{*}\left(data\right)$.

The mutability μ(data) of the entire file data.txt is simply given by

(13) $$\mu \left(data\right)=\frac{w^{*}\left(data\right)}{w(data)}.$$

Similarly, the diversity of the file data.txt is also a ratio, namely,

(14) $$\delta \left(data\right)=\frac{\lambda^{*}\left(data\right)}{\lambda (data)}.$$

The previous definitions can also be dynamic at time *t* and given with respect to the ν records in data.txt counted from that one at time *t*. Then, we can define the dynamic parameters for data.txt:

(15) $$\mu \left(t,\nu \right)=\frac{w^{*}\left(t,\nu \right)}{w(t,\nu )}$$

and

(16) $$\delta \left(t,\nu \right)=\frac{\lambda^{*}\left(t,\nu \right)}{\lambda (t,\nu )}.$$

**Figure 1:**
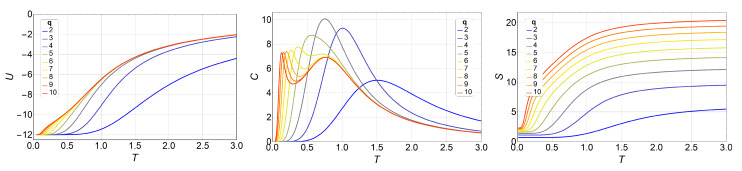
Internal energy *U* (left), specific heat *C* (middle), and entropy *S* (right) of the *q*-state clock model as a function of temperature *T* for a 3×3 lattice without a magnetic field for different values of *q*. From q=2 (blue) to q=10 (red).

As shown in the discussion accompanying Figure 1 of Reference [25], referred to the Ising model, mutability can be more appropriate for discussing the variability of a parameter with changing conditions (like an increase in temperature), while diversity is more appropriate for discussing critical phenomena, such as phase transitions. This is one of the issues discussed below in light of the results obtained from the clock model.

## 3. Results and Discussions

Let us begin by considering the theoretical approach to the *q*-state clock model according to Equation (2) for B=0, considering an L=3 lattice, as presented in the previous section. We successfully calculated the partition functions from q=2 (Ising) to q=10. From here, all the thermodynamic quantities of the system can be analytically obtained as a function of the temperature *T*.

Figure 1 shows the results obtained for internal energy U(T), specific heat C(T), and entropy S(T).

For the specific heat, *C* vs. *T* (center of Figure 1), we see that the two peaks appear for q≥6. At q=5, we see only one peak, but it is notoriously more skewed to the right. We will see that this is due to the small size of the lattice. Therefore, for small systems, the BKT phase appears only for q≥6.

**Figure 2:**
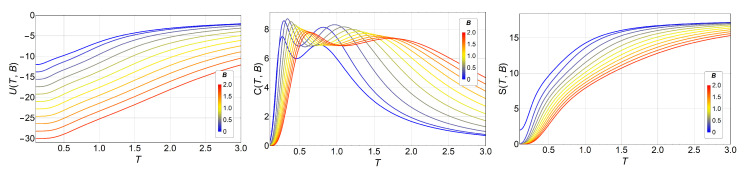
Internal energy U(T,B) (left), specific heat C(T,B) (middle), and entropy S(T,B) (right) of the *q*-state clock model (q=7) as a function of temperature *T* and magnetic field *B* for a 3×3 lattice. Different magnetic fields *B* are indicated by lines of different colors, from B=0 (blue) to B=2 (red).

We can see here (Figure 1, right) the basic features of entropy for the low- and high-temperature limits. When no external field is applied, the energy ground state of the ferromagnetic *q*-state clock model has a degeneracy equal to *q*, independent of the total number of spins *N*; therefore, S(0)=lnq. On the other hand, at very high temperatures, the exchange interaction is overridden, and every spin has *q* degrees of freedom with equal probabilities: hence, the system degeneracy is equal to $q^{N}$, and, therefore, Figure 2 shows the same observables in the presence of magnetic field *B*, namely, U(T,B), C(T,B), and S(T,B), for the case where q=7, as an example. The shift of the transition temperatures due to the variations in the magnetic field is clearly visible. This is also an exact analytical result obtained by calculating straightforwardly the partition function of Equation (3). The magnetic field was applied along the (1,0) direction, along which a spin orientation is always possible, regardless of the *q* value.

For B>0, the Zeeman term is added to the energy (see Equation (2)); therefore, the FM ground state energy is lowered by the external field. As an example for B=2, the Zeeman energy is −18, and this is added to the ground state energy (−12) at zero field; therefore, the new ground state energy is −30 (red curve in the left of Figure 2).

We also observe that the shapes of specific heat curves, *C* vs. *T*, maintain the two peaks, but they shift to higher temperatures as the external field goes from B=0 to B=2. This behavior is easily explained by the external field favoring ordered phases (FM and BKT) over disordered ones, and, therefore, the transition temperatures increase with the strength of the external field.

The magnetic field breaks the degeneracy of the ground state so that S(0)=0 for B≠0 instead of S(0)=lnq. However, at high temperatures, the Zeeman term in Equation (2) is overridden, and we are back to the same situation as in previous analysis for B=0, namely, every spin has *q* degrees of freedom with equal probabilities: hence, the system degeneracy is equal to $q^{N}$, and, therefore, the entropy S(T>>J)=Nlnq, as observed in the right of Figure 2.

### 3.1. Monte Carlo Simulations

**Figure 3:**
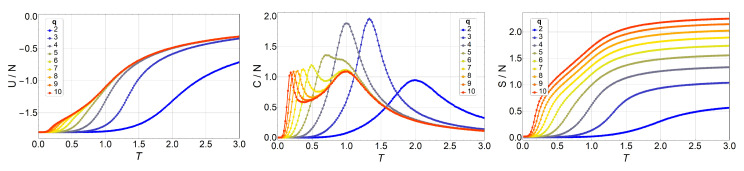
Internal energy U/N (left), specific heat C/N (middle), and entropy S/N (right) of the *q*-state clock model as a function of temperature *T* for an N=10×10 lattice without a magnetic field for q=2 (blue) to q=10 (red).

**Figure 4:**
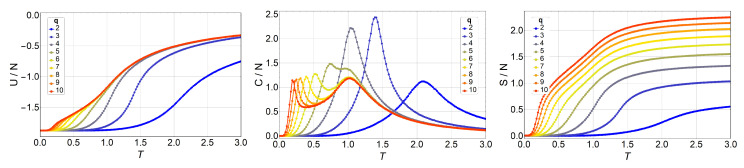
Internal energy U/N (left), specific heat C/N (middle), and entropy S/N (right) of the *q*-state clock model as a function of temperature *T* for an N=16×16 lattice without magnetic field for q=2 (blue) to q=10 (red).

**Figure 5:**
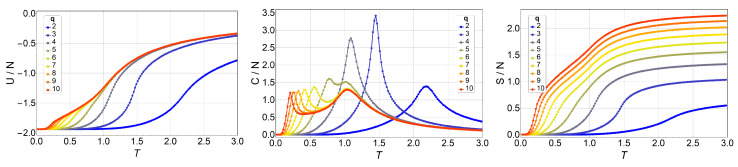
Internal energy U/N (left), specific heat C/N (middle), and entropy S/N (right) of the *q*-state clock model as a function of temperature *T* for a N=32×32 lattice without magnetic field for q=2 (blue) to q=10 (red).

**Figure 6:**
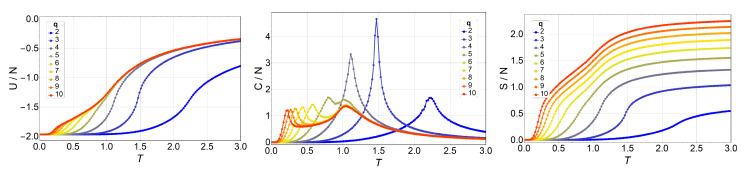
Internal energy U/N (left), specific heat C/N (middle), and entropy S/N (right) of the *q*-state clock model as a function of temperature *T* for a N=64×64 lattice without magnetic field for q=2 (blue) to q=10 (red).

Next, we present and discuss the output from Monte Carlo simulations made for the *q*-state clock model in lattices of up to 64×64 with free boundary conditions. We began by simulating a 3×3 lattice for different *q* values and comparing these numerical results to the analytic ones presented in the previous section. We did not find a single difference, which is expected of course, but which also serves as a check for the computer programs used extensively in the simulations reported next. Thus, thermodynamic observables for lattices 10×10, 16×16, 32×32, and 64×64 are presented in Figure 3, Figure 4, Figure 5 and Figure 6, respectively.

We observe an overall self-agreement in the shape of energy *U*, specific heat *C*, and entropy *S* as functions of temperature for the different lattices. For a given size, the peak of the specific heat occurs at a lower temperature as *q* increases, and then it splits in two peaks. As *q* continues to increase, the high-temperature peak remains at the same temperature, whereas the low-temperature peak tends toward lower temperatures (eventually to a temperature of zero) as *q* increases tending to infinity. This low-temperature peak, $T_{1}$, is the transition from the FM phase to the BKT-like phase, which is characterized by vortex spin configurations and FM spin–spin correlated configurations, like waves. Both the vortex and spin waves are low-energy excitation that occurs as *q* increases. Therefore, for q→∞, we expect that $T_{1}\to 0$. We would like to stress the size-dependence of the peak heights at fixed *q*. Indeed, for q=2,3, and 4, the unique peak corresponds to a second-order phase transition, hence, the specific heat C/N is expected to diverge with *N* at $T=T_{1}$. However, for q≥5, the two peaks should correspond to two BKT transitions, which are infinite-order; that is, there is no divergence in C/N at $T_{1}$ and $T_{2}$. Both features are observed in Figure 3, Figure 4, Figure 5 and Figure 6, and, furthermore, the constant height of the peak at $T=T_{2}$ for q=5 could represent further evidence of a BKT-like transition. To be more specific on the characterization of the clock model, we discuss next a phase diagram including the three possible magnetic phases: FM (long-range order), BKT (short-range correlations), and PM (total disorder).

### 3.2. Phase Diagram

**Figure 7:**
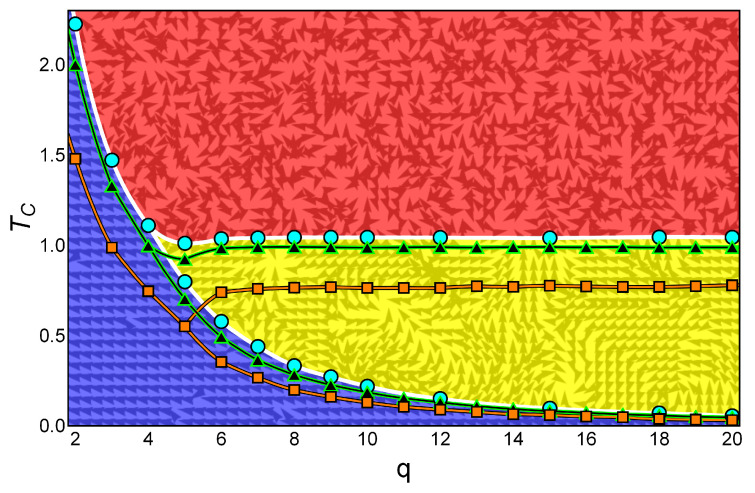
Phase diagram for the *q*-state clock model for a 3×3 lattice (squares), a 10×10 lattice (triangles), and a 64×64 lattice (circles) with free boundary conditions. For temperatures under $T_{1}$, the ferromagnetic (FM) ordered phase dominates. For q<5, the transition from this FM ordered phase is to the disordered PM phase. For large enough lattices and q≥5, the transition from the FM phase is to the partially ordered Berezinskii–Kosterlitz–Thouless (BKT) phase. Then, a transition at a higher temperature $T_{2}$ appears which separates the BKT phase from the paramagnetic (PM) phase; it can be noticed that $T_{2}$ is essentially constant with respect to *q*. The background colors blue, yellow, and red (from bottom to top) mark the FM, BKT, and PM phases, respectively. Spin orientations for one possible snapshot corresponding to the 64 × 64 lattice and q=9 are given in the gray color over the background colors.

In this section, we show the phase diagram for the clock model as extracted from the specific heat. We collected the analytic results for the 3×3 lattice and the numerical results for the 10×10 and the 64×64 lattices. This is shown in Figure 7 for *q* ranging from 2 to 20 using squares, triangles, and circles, respectively. The texture and color underneath illustrate the instantaneous magnetic phases for the larger lattice and q=9 just as examples for the kind of magnetic ordering present in each phase.

Several features in Figure 7 deserve special attention. First, the lower critical temperature $T_{1}$ follows a monotonous decrease with *q* approaching zero asymptotically. Second, the higher critical temperature $T_{2}$ remains at a constant value for q≥6. Third, for q=5, there is just one critical temperature following the tendency of $T_{1}$, but for L=10 (and also for L=64), the two transitions are clearly visible for q=5. Fourth, an order parameter beyond the usual magnetization is necessary to distinguish the BKT phase from the PM disordered phase.

**Figure 8:**
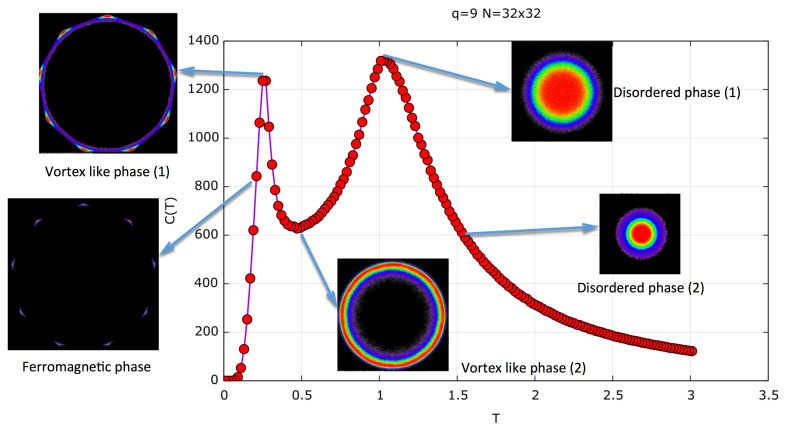
Specific heat for q=9 in a 32×32 lattice (red curve). The figures depict the 2D order parameter distribution of *M* (two-component vector), as defined in the text, for T=0.21,0.27,0.5,1, and 1.51. In the ferromagnetic (FM) phase, the distribution presents a nine-fold symmetry, indicating the nine possible orientations of the magnetic domains in the FM phase at T=0.21. Vortex-like phase (1) shows (T=0.27) the onset of the BKT phase where we have smaller FM domains and vortex structures. This makes the thermal average magnetization (the 2D order parameter) rotate while keeping its overall magnitude. At T=0.27, the FM phase is seemingly still present, as can be seen in the ring structure with nine maxima, as observed at T=0.21. In T=0.5, a pure BKT phase is observed as a uniform ring structure, but their radii are beginning to shrink, maintaining a smaller magnitude. At T=1 is the onset of the disordered phase in which the magnitude of the magnetization decreases, filling the interior of the circle as a 2D Gaussian distribution with a zero average. At T=1.51, the overall magnitude of the *M* parameter further shrinks. The color code of the 2D order parameter distribution goes from black to purple, blue, green, yellow, and red as values of the 2D distribution increase. The radii of the ring-like 2D distributions represent the thermal magnetization modulus as a function of temperature, and this is given in Figure 10.

To further specify the different phases, Figure 8 shows the specific heat for a 32×32 lattice as a function of temperature. In this figure, we show a two-dimensional (2D) order parameter at certain characteristic temperatures which clearly discriminate the three (FM, BKT, and PM) different phases. The order parameter we use is the 2D distribution of the variable $M=(M_{x},M_{y})$, as defined in Equation (7), that is, the spin-lattice average at time *t* after the thermalization process of τ MCSs.

This corresponds to the vector spin average for a given spin configuration at time *t*. This vector is then calculated after the thermalization, and every 20 MCSs of a total of $N_{c}$ times. By plotting all $N_{c}$ vectors, we generate a 2D distribution that clearly characterizes the different phases. In the FM phase, only certain directions of the spin are allowed. In Figure 8, constructed for q=9, we see that at low temperature, the average magnetization vector points in nine directions which correspond to the nine-fold symmetry of the FM states with equal probability. The BKT-like phase is characterized by spin waves and vortex structures; therefore, the lattice average of the spin points to any of the 2π directions while conserving, to a great extent, its magnitude in every lattice average. Therefore, a ring structure is formed. In the disordered (PM) phase, every spin in the lattice points randomly to any direction; therefore, the lattice average magnetization distribution exhibits a 2D-Gaussian peak with decreasing magnitude as *T* increases; hence, the circle begins to be filled with a higher probability (red color) near the center. The color code goes from black (zero value) to purple, blue, green, yellow, and red in increasing order of probability for this 2D order parameter. This parameter was already introduced as a complex order parameter by Baek et al. [31].

**Figure 9:**
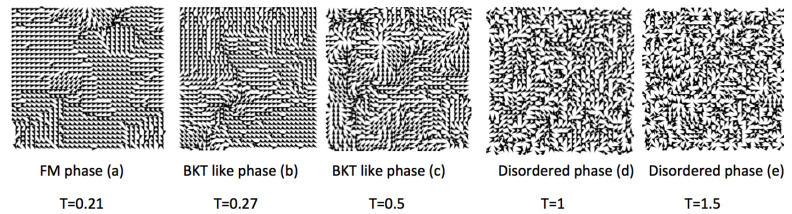
Spin arrangements of the *q*-state clock model for q=9 in a 32×32 lattice. The figure depicts five snapshots, where each one is a sample of the $2\times 10^{5}$ states used for thermal averages at different temperatures. The temperatures are the same as those shown in Figure 8, i.e., T=0.21,0.27,0.5,1, and 1.51. The snapshots clearly show the FM phase (**a**), the BKT-like phase (**b**) and (**c**), and the disordered phase (**d**) and (**e**).

The next figure (Figure 9) depicts snapshots of some spin configurations showing the spin arrangements that occur at different temperatures.

**Figure 10:**
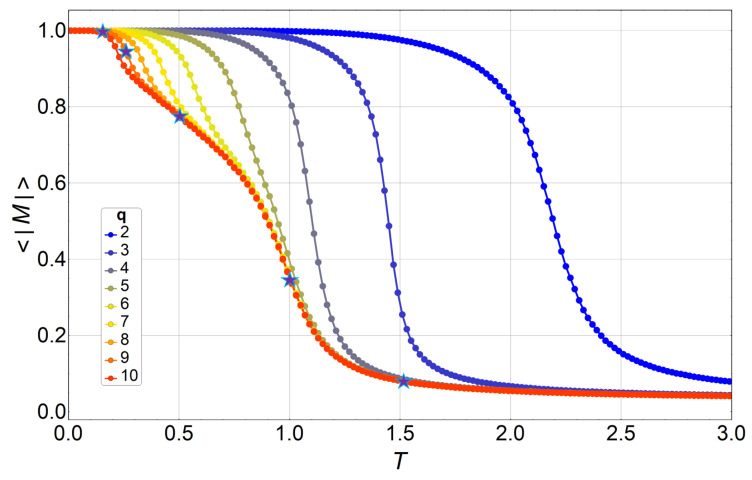
Thermal average of the absolute value of the magnetization, as defined in Equation (8), for q=2 (blue) to q=10 (red) in a 32×32 lattice. The figure also depicts five stars for q=9 at the temperatures where the 2D order parameter is shown in Figure 8 for T=0.21,0.27,0.5,1, and 1.51. The values of their absolute magnetization are the average radii of the ring and circular point distributions shown in Figure 8.

The next figure (Figure 10) shows the magnetization modulus as the thermal average of the spin-lattice average, as defined in Equation (8).

Let us now consider the information content as an independent test to characterize these phase transitions. We shall concentrate on the simulations for 32×32 lattices, measuring μ and δ as defined in the Models and Methods Section.

**Figure 11:**
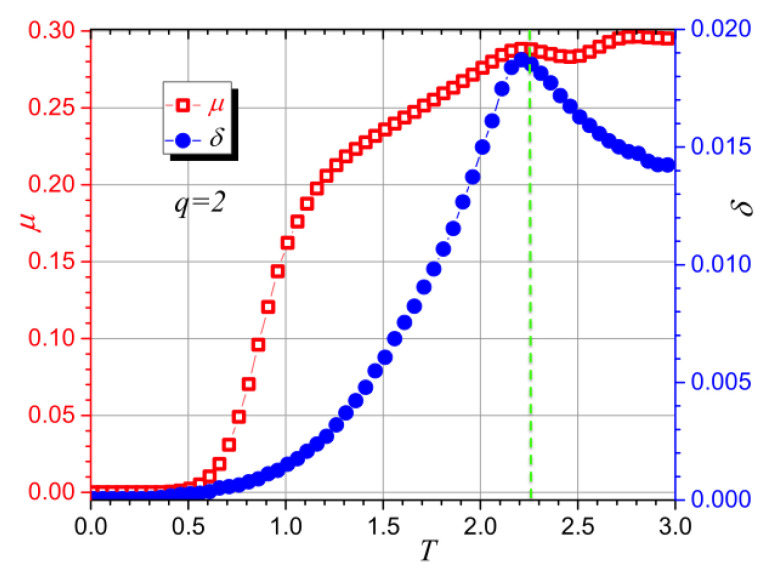
Mutability (μ) and diversity (δ) for the energy data for L=32, q=2. The vertical dashed line corresponds to the position of the Onsager solution valid for the thermodynamic limit.

Figure 11 presents the information content results for the same energy series generated by the previously described MC algorithm; the case of a 32×32 lattice for q=2 was chosen for this report. The open-squares curve (red) gives the results for mutability, and it presents a local maximum near T=2.2, in agreement with the specific heat results (see Figure 5). The solid-circles curve (blue) is the result for diversity and shows a sharper absolute maximum at T=2.2. The vertical dashed line illustrates the temperature 2.269, at which the Onsager solution predicts the transition for an extremely large system [32]. This is just a reference since our systems are finite and have free boundary conditions, so they do not correspond well with this theoretical solution.

The previous results were obtained from sequences presenting energy data. However, there are more sensitive parameters associated with states of the system whose sequences present alternatives which can recognize this phase transition in a better way. These are the cases of the magnetic order parameters (magnetization, neighbor correlations, site-memory correlation) [25] which we initially tested and obtained encouraging results. To consider this additional study is beyond the goals of the present paper, so we will stick to the energy data results analyzed for diversity to directly compare with the results reported above.

**Figure 12:**
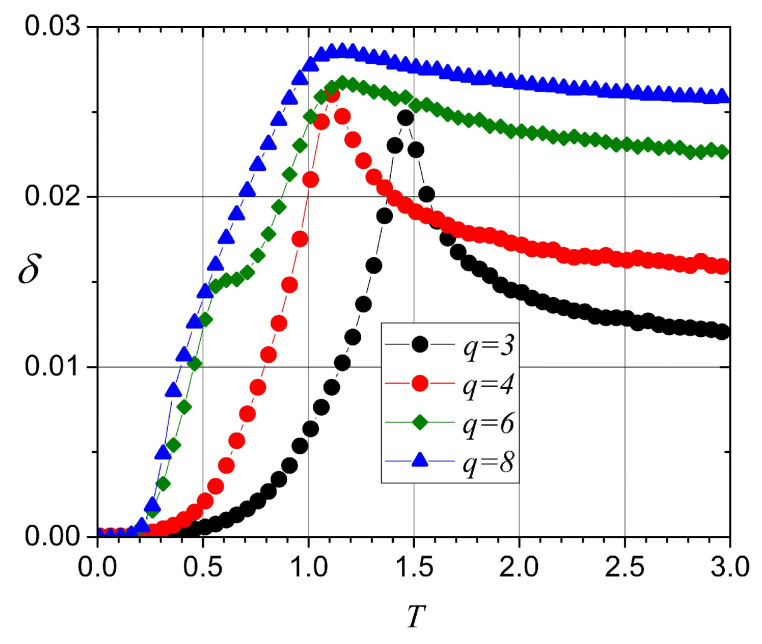
Diversity δ of the energy data for L=32, q=3, q=4, q=6, and q=8.

In Figure 12, we present the results for δ in the cases for q=3, q=4, q=6, and q=8. The ordinates were multiplied by the appropriate constants to fit a common arbitrary scale since the meaningful information is in the temperatures that are marked by each maximum or the inflections of the curves. For q=3, just one critical temperature is visible and in agreement with the maximum of the specific heat for this lattice size, as shown in Figure 5. A similar situation is observed for q=4, with a maximum at a lower temperature of around 1.1. For q=6, a maximum of around 1.1 and a “knee” just over 0.5 are visible, in agreement with the two maxima for this value of *q* reported in the figure for specific heat. In the case of q=8, the maximum near 1.1 is clearly present, although it is a bit broader than that for q=6. The “knee” is a barely visible tiny change in slope at T=0.5.

The results shown by the previous two figures, as well as similar ones for other intermediate values of *q*, show that the information method is able to recognize the transitions present in the clock model for low values of *q* when applied to the energy data vectors produced by the MC simulations. However, the phases are less detectable by this method as *q* increases.

The previous shortcomings of the information theory method could be due to the system itself and the way in which the transition is characterized. With respect to the former, it should be noticed that as *q* increases, the number of possible configurations increases, the energy intervals decrease, and the density of states is highly degenerate and tending to a continuum as q→∞; to distinguish among energy values is now increasingly difficult. With respect to the latter, energy is not the best order parameter to characterize these transitions, and, eventually, different order parameters more oriented to the recognition of magnetic states of the system (magnetization, site memory, neighbor correlations) can render better results. At the moment, this is an open question, and it should be addressed in future work.

## 4. Conclusions

Using analytically derived expressions and Monte Carlo simulations, we explored the *q*-state clock model for square lattices with free boundary conditions which better mimic the properties of small systems for which this approach is intended. We calculated their thermodynamic properties and characterized the three magnetic phases present for this model. The corresponding magnetic phase diagrams were calculated for lattice sizes of up to 64×64. It turns out that there exists an FM below a critical temperature $T_{1}$. For q≥5 and lattice sizes over 10 × 10, a BKT-like phase appears for temperatures between $T_{1}$ and a second critical temperature $T_{2}$, separating this BKT phase from the disordered paramagnetic phase. The BKT phase reflects partial magnetic ordering characterized by spin vortex configurations and zones with FM spin–spin correlation, reflecting curling, or wave-like ordering. The three phases can be well characterized using the spin-lattice average distribution. This 2D distribution shows characteristic patterns which clearly identify the three (FM, BKT, and PM) phases.

The entropy of the system always increases with temperature, showing subtle slope changes at the transition temperatures $T_{1}$ and $T_{2}$. The low-temperature limit for the entropy is simply given by ln(q) in absence of a magnetic field, while it vanishes for any magnetic field that breaks the degeneracy of the ground state, yielding a singlet as a ground state. On the other hand, the high-temperature value for entropy tends asymptotically to Nln(q), thus reflecting that all degrees of freedom are equally probable.

The information theory method produces results that are in agreement with those of the specific heat of the system. It distinguishes the phase diagram presented in Figure 7 for low values of *q*, thus confirming previous results obtained by conventional treatments. However, this recognition is progressively lost as *q* increases. This is probably due to the fact that the information recognition points to the energy values which are shared by increasingly more states as *q* increases (contribution of the internal degrees of freedom to the density of states). Eventually, different simulations involving a more elaborated order parameter are needed to address the slight difference between the BKT phase and the disordered phase, both with vanishing magnetization. This work is in progress and should produce results in the near future.

## Figures and Tables

**Table 1:** Coefficients $c(E_{n},q)$ for a 3×3 lattice with free boundary conditions and B=0 for q=4 and q=6. The first column enumerates energy levels, *n*, and the second and third columns give the corresponding energies *E* and degeneracies c(E,4), respectively. The fourth and five columns show half of the energies and degeneracies for q=6, respectively. The rest of the energies and degeneracies can be found using the following symmetry: $c\left(-E_{n},6\right)=c\left(E_{n},6\right)$.

*n*	q=4			q=6	
	$E_{n}$	$c(E_{n},4)$		$E_{n}$	$c(E_{n},6)$
1	−12	4		−12	6
2	−10	32		−11	48
3	−9	128		−10.5	192
4	−8	248		−10	348
5	−7	896		−9.5	960
6	−6	2336		−9	2448
7	−5	4864		−8.5	2736
8	−4	10,748		−8	5376
9	−3	19,712		−7.5	11,808
10	−2	29,376		−7	14,880
11	−1	39,936		−6.5	22,128
12	0	45,584		−6	54,072
13	1	39,936		−5.5	54,960
14	2	29,376		−5	94,032
15	3	19,712		−4.5	175,968
16	4	10,748		−4	191,514
17	5	4864		−3.5	231,744
18	6	2336		−3	478,752
19	7	896		−2.5	393,360
20	8	248		−2	530,892
21	9	128		−1.5	806,736
22	10	32		−1	707,760
23	12	4		−0.5	701,712
24				0	1,112,830

**Table 2:** Coefficients $c(E_{n},5)$ for a 3×3 lattice with free boundary conditions and B=0. In this case, there are no symmetries and, therefore, we present all $E_{n}$ values with their respective degenerations, from n=1 to n=85. Here, as explained in the manuscript, the symmetry $S_{i}\to -S_{i}$ is not present.

*n*	q=5										
	$E_{n}$	$c(E_{n},5)$	*n*	$E_{n}$	$c(E_{n},5)$	*n*	$E_{n}$	$c(E_{n},5)$	*n*	$E_{n}$	$c(E_{n},5)$
1	−12	5	23	−4.92705	15,760	45	−1.57295	15,760	67	2.30902	89,000
2	−10.618	40	24	−4.76393	290	46	−1.47214	4100	68	2.47214	1340
3	−9.92705	160	25	−4.66312	6720	47	−1.30902	124,320	69	2.73607	58,080
4	−9.23607	290	26	−4.5	8960	48	−1.1459	1680	70	3	30,200
5	−8.54508	800	27	−4.23607	30,360	49	−1.04508	27,920	71	3.16312	6720
6	−8.38197	40	28	−4.07295	680	50	−0.881966	57,000	72	3.42705	64,120
7	−8.11803	320	29	−3.97214	5280	51	−0.618034	117,040	73	3.8541	21,160
8	−7.8541	1680	30	−3.80902	23,080	52	−0.454915	6958	74	4.11803	23,640
9	−7.42705	680	31	−3.7082	410	53	−0.354102	9200	75	4.28115	1440
10	−7.16312	1600	32	−3.54508	29,120	54	−0.190983	124,320	76	4.54508	27,920
11	−7	39,936	33	−3.38197	5800	55	0	2	77	4.97214	5280
12	−6.73607	3040	34	−3.28115	1680	56	0.072949	64,120	78	5.23607	17,720
13	−6.57295	160	35	−3.11803	57,000	57	0.236068	30,360	79	5.66312	9880
14	−6.47214	1340	36	−2.95492	800	58	0.5	147,600	80	6.09017	560
15	−6.30902	1760	37	−2.8541	21,160	59	0.663119	1600	81	6.3541	9200
16	−6.04508	6960	38	−2.69098	23,080	60	0.763932	17,720	82	6.78115	1680
17	−5.88197	320	39	−2.59017	1240	61	0.927051	77,360	83	7.47214	4100
18	−5.78115	1440	40	−2.42705	77,360	62	1.19098	89,000	84	8.59017	1240
19	−5.61803	5800	41	−2.26393	3040	63	1.3541	8440	85	9.70820	410
20	−5.3541	8440	42	−2.16312	9880	64	1.61803	117,040			
21	−5.19098	1760	43	−2	67,600	65	1.88197	23,640			
22	−5.09017	560	44	−1.73607	58,080	66	2.04508	29,120			
